# **A**ssessing ECG Findings in Pediatric COVID-19 Patients: A Comprehensive **Analysis**

**DOI:** 10.2174/011573403X354415250727070214

**Published:** 2025-07-31

**Authors:** Mohammadreza Naghibi, Shirin Sadat Ghiasi, Rasoul Raesi, Feisal Rahimpour

**Affiliations:** 1 Pediatric Interventional Cardiology, Pediatric and Congenital Cardiology Division, Pediatric Department, Faculty of Medicine, Mashhad University of Medical Sciences, Mashhad, Iran;; 2 Faculty of Medicine, Mashhad University of Medical Sciences, Mashhad, Iran;; 3 Department of Nursing, Torbat Jam Faculty of Medical Sciences, Torbat Jam, Iran;; 4 Department of Health Services Management, School of Health, Mashhad University of Medical Sciences, Mashhad, Iran;; 5 Department of Pediatric, Interventional Electrophysiologist, Mashhad University of Medical Sciences, Mashhad, Iran

**Keywords:** Electrocardiogram, pediatric, COVID-19, myocardium, heart failure, S wave fragmentation

## Abstract

**Introduction:**

COVID-19 can be associated with varying degrees of cardiac involvement in children, such that myocardial damage can be caused directly by the COVID-19 virus itself or systemic inflammation caused by the infection. The present study was conducted with the aim of evaluating ECG findings in children with COVID-19.

**Methods:**

This is a prospective cross-sectional study that was conducted by census method on 764 children with COVID-19 in hospitals related to Mashhad University of Medical Sciences in 2022. The data were extracted using a checklist including clinical information and medical records of the patients and analyzed using descriptive and statistical tests.

**Results:**

764 children with COVID-19 were examined, of which 385 (51%) were male. The studied patients included MISC (25.9%), Kawasaki-like disease (0.3%), pulmonary (12.7%), and gastrointestinal (2%) involvements. More than half of the patients (58%, 444 patients) showed changes in echocardiography findings, including mitral valve insufficiency, dilation of one or more cardiac chambers, and pericardial effusion. 98.8% of patients had NAX. AVB grade I was found in 26 patients (3.4%). Abnormal ST-T segments were observed in 25 patients (3.3%). The prevalence rate of S wave fragmentation was 2.8% (21), and fragmented R waves were found in 13 patients (1.7%).

**Discussion:**

Patients hospitalized in the intensive care unit (ICU) had a higher amount of disorder for each parameter change. Additionally, a significant association was found between the higher occurrence of AV node block and arrhythmia with clinical status (*p*<0.05), with the same higher rate in patients kept in the ICU.

**Conclusion:**

ECG findings can be used to predict the presence or absence of myocardial involvement as well as its severity. Furthermore, patients with changes in ST-T fQRS and PR interval are more likely to experience cardiac involvement, which could result in a poorer prognosis.

## 1 INTRODUCTION

During the COVID-19 pandemic, varying degrees of cardiac involvement have been observed in children, ranging from mild pericardial inflammation to more severe conditions such as coronary artery aneurysms, cardiomyopathy, and even death [1-3]. While echocardiography is the preferred diagnostic method for evaluating the heart, it may not be readily available in all healthcare settings and can impose a significant socioeconomic burden on the healthcare system [4, 5]. As a result, electrocardiograms (ECGs) have emerged as a simple and accessible screening tool for COVID-19 patients [6, 7]. The novel coronavirus, which was first reported in China in December 2019, has rapidly spread across the globe and was declared a pandemic by the World Health Organization [5, 8]. It has been well-documented that COVID-19 impacts the respiratory and gastrointestinal tracts, and also has negative effects on the cardiovascular system [8-10]. Previous reports suggest that myocardial damage can be caused directly by the virus itself or systemic inflammation [10, 11]. Moreover, people with a history of cardiovascular disease are more susceptible to severe acute respiratory syndrome coronavirus 2 (SARS-CoV-2). Several studies have shown that COVID-19 is more lethal in individuals who have had heart damage during the time of infection or who have a previous history of heart disease [12, 13]. Therefore, it is crucial to predict the negative effects of SARS-CoV-2 infection on the cardiovascular system and detect myocardial damage early on [14, 15].

Several studies have demonstrated a link between myocardial scarring caused by coronary artery disease (CAD) and the presence of fQRS. The presence of fragmented waves (fQRS) within the QRS complex on a standard electrocardiogram (ECG) indicates a ventricular conduction disorder and suggests heart damage caused by other diseases [16-18]. The location of fQRS on the ECG is strongly correlated with the location of the scar in the left ventricle [19, 20]. Compared to other ECG ischemia markers, fQRS is more sensitive in detecting myocardial fibrosis by SPECT imaging in patients with CAD. ECG is an inexpensive and user-friendly diagnostic tool that poses a lower risk of infection transmission compared to other methods like transesophageal echocardiography and MRI, especially during a pandemic [21, 22]. Detection of fQRS on ECG can facilitate early, practical, and safe diagnosis of myocardial damage during the COVID-19 period [19, 23, 24]. Early detection of cardiac involvement in children can significantly improve clinical outcomes for this patient group. While echocardiography remains the preferred method for heart assessment, its limited availability and high costs make ECG a crucial tool for rapid, accessible, and cost-effective screening. Given the current lack of sufficient data regarding ECG features in children with COVID-19 and their link to clinical outcomes, coupled with the fact that children may not always present with obvious clinical signs of cardiac involvement, utilizing ECG can help identify hidden cases and prevent more serious complications. Furthermore, most existing studies have focused on small cohorts or lacked correlation with clinical outcomes and echocardiographic findings. This gap is critical because children often present with subtle or atypical cardiac symptoms, increasing the risk of underdiagnosis and delayed intervention. Through a comprehensive examination of ECG changes in children with COVID-19, this study aims to pinpoint specific patterns indicative of cardiac involvement, facilitating early diagnosis and timely intervention. This study aimed to investigate ECG findings in children with COVID-19.

## 2 MATERIALS AND METHODS

### 2.1 Study Design

This prospective cross-sectional study was conducted using the census method on 764 children under the age of 18 diagnosed with COVID-19. The study took place in hospitals affiliated with Mashhad University of Medical Sciences in 2022. The decision to include pediatric patients under 18 years of age was based on two key considerations: the age range aligns with previous relevant studies [25], and in our institution and regional practice, conventional pediatric services are typically provided to patients up to the age of 18.

### 2.2 Inclusion and Exclusion Criteria

The inclusion criteria for this study comprised pediatric patients under 18 years of age diagnosed with COVID-19, confirmed *via* RT-PCR and imaging. Patients with negative COVID-19 results were excluded due to the small sample size, and those with incomplete or invalid data were also excluded to ensure the reliability of the findings.

### 2.3 Data Collection

COVID-19 testing was performed according to WHO protocol, with nasopharyngeal swabs collected from all patients. RNA SARS-CoV-2 was detected by reverse transcription polymerase chain reaction (RT-PCR) and confirmed by chest X-ray and computed tomography. Demographic variables (age and sex), clinical data, and laboratory tests were recorded in hospital files. The presence of any ECG abnormality was defined as the presence of one or more of the following abnormalities: non-sinus rhythm, atrioventricular block, QRS ≥ 90 ms, ST changes, negative T wave, cQT ≥ 440, pathologic Q/QS, or the presence of PAC or PVCs. Prognosis, mortality rate, number of hospitalization days, and clinical condition at the time of discharge were recorded. In this study, QT intervals were measured manually from the onset of the QRS complex to the end of the T wave. The corrected QT (QTc) interval was calculated using Bazett’s formula (QTc = QT / √RR), a widely accepted method for QT [26]. In this study, all ECGs were performed using standardized 12-lead ECG machines with pediatric-specific settings, in accordance with the guidelines recommended by the American Heart Association (AHA), which emphasize the importance of high sampling frequency, appropriate filter settings, and accurate interpretation of ECGs in children. These standards are outlined in international guidelines for recording and interpreting ECGs in pediatric populations. Additionally, all ECG interpretations in this study were conducted by a pediatric cardiologist and arrhythmia specialist.

### 2.4 Data Analysis

Mean and standard deviation were reported for continuous variables, while numbers and ratios were reported for discrete variables. Continuous variables were compared using analysis of variance, while ratios were compared using Fisher's exact test or chi-square test. Multivariate regression was used for discrete variables, and a generalized linear model was used for continuous variables. ECG variables were adjusted for clinical and laboratory variables that showed a statistically significant or borderline difference (*p* ≤ 0.1) between the two groups.

The analysis consisted of two parts: descriptive and analytical. Prevalence and median quartiles for numerical variables were reported, with all variables having a normal distribution. Analytically, variables were first compared between the positive and negative groups.

## 3 RESULTS

### 3.1 Demographic and Clinical Characteristics of Patients

A total of 764 pediatric patients with COVID-19, with a median BMI of 18.3 ± 18.07, were referred for cardiovascular evaluation. Among these, 385 (51%) were male, and 379 (49%) were female. The majority of patients (94.4%) were admitted to the general ward, while 3.7% were in the ICU, and 1.9% were outpatients. Based on clinical criteria, patients were classified into the following categories: Multisystem Inflammatory Syndrome in Children (MISC) (25.9%), Kawasaki-like disease (0.3%), pulmonary involvement (12.7%), and gastrointestinal involvement (2%). Additionally, 59.1% of patients with positive COVID-19 PCR results were clinically asymptomatic (Table **1**).

### 3.2 Laboratory and ECG Findings

Laboratory findings revealed that CKMB levels were greater than 24 u/liter in 10 patients (1.3%), and TPi was positive in 11 patients (1.5%). High D-dimer levels (>500) were observed in 63 patients (78.8%). ECG abnormalities included normal axis (NAX) in 98.8% of patients, right axis deviation (RAD) in 0.7%, left axis deviation (LAD) in 0.1%, right conduction delay in 0.1%, and left conduction delay in 0.3%. First-degree AV block (AVB) was found in 26 patients (3.4%), while second-degree AVB and complete heart block (CHB) were observed in 1 and 2 patients, respectively. Abnormal ST-T segments were noted in 25 patients (3.3%). Fragmented S waves were observed in 21 patients (2.8%), and fragmented R waves were found in 13 patients (1.7%) (Table **2**).

### 3.3 Echocardiography Findings

More than half of the patients (58%, 444 patients) showed changes in echocardiography findings, including mitral valve insufficiency, dilation of one or more cardiac chambers, and pericardial effusion. These changes were more prevalent in patients admitted to the ICU (Table **3**).

### 3.4 Association of Findings with Disease Severity and Clinical Status

No significant correlation was found between age or gender and disease severity. However, a significant relationship was observed between general health condition and prolonged PR interval, as well as increased QRS duration (*p* < 0.05). Patients hospitalized in the ICU had a higher prevalence of ECG abnormalities, including AV node block, arrhythmias, ST-T changes, and fragmented S and R waves (*p* < 0.05). Echocardiographic changes were also significantly associated with ICU admission (*P* = 0.02) (Table **4**).

Table **5** presents the distribution of various arrhythmias observed in pediatric COVID-19 patients. Sinus tachycardia was the most common arrhythmia, occurring in 96 patients (12.7%), likely reflecting the body's stress response to infection or inflammation. Atrioventricular block (AVB) was observed in 29 patients (4.2%), suggesting potential conduction system abnormalities due to myocardial inflammation or ischemia. Sinus bradycardia, though less common, was noted in 10 patients (1.3%), possibly indicating autonomic dysfunction or direct effects on the sinoatrial node. Premature atrial contractions (PAC) and premature ventricular contractions (PVC) were rare, each occurring in 3 patients (0.4%), which may point to minor myocardial irritation or electrolyte imbalances. Supraventricular tachycardia (SVT) and atrial tachycardia (AT) were very rare, with SVT observed in 1 patient (0.1%) and AT in 2 patients (0.3%), indicating occasional severe electrical instability in the atria. Early repolarization, sinus arrhythmia, atrial rhythm, and atrial parasystole were also rare (≤0.3%), with early repolarization and sinus arrhythmia typically being benign findings.

Table **6** presents the distribution of S-wave and R-wave fragmentations observed on electrocardiograms (ECGs) in pediatric COVID-19 patients. For S-wave fragmentation, the majority of patients (724, 97.2%) had normal findings, while minor fragmentation was observed in specific leads: lead I (2 patients, 0.3%), lead III (1 patient, 0.1%), lead V (5 patients, 0.7%), and combinations of leads such as I/II (1 patient, 0.1%), II/III (6 patients, 0.8%), I/II/III (2 patients, 0.3%), and others (each ≤0.1%). Similarly, for R-wave fragmentation, 735 patients (97%) had normal findings, with minor fragmentation observed in lead II (2 patients, 0.3%), lead III (1 patient, 0.1%), lead V (3 patients, 0.4%), and combinations of leads such as III/IVL (2 patients, 0.3%), aVL/IVF (1 patient, 0.1%), III/LVF (1 patient, 0.1%), and II/III/I/AVF (3 patients, 0.4%).

## 4 DISCUSSION

This study aimed to investigate ECG findings in children with COVID-19. Electrocardiography is a widely used non-invasive diagnostic procedure that provides rapid results for evaluating cardiovascular health, particularly for diagnosing myocardial ischemia and cardiac arrhythmia. A basic ECG was conducted before treatment and during follow-up to assess the impact of COVID-19 severity on ECG parameters, including arrhythmia, blocks, ST-T changes, and the presence of fQRS. Upon examining changes in ECG waveforms in our COVID-19 patients, we noted that the PR interval, which refers to increased conduction time, indicated a first-degree AV block. We observed that AV block, a disorder of the conduction pathway from the SA node to the ventricle, was most commonly seen in cases of atrial myocardial inflammation, while changes in other ECG parameters were similar to those observed in the general population. Our study revealed a significant correlation between an increased PR interval and disease severity in our population, the majority of whom were hospitalized in the ICU. Hassan *et al*. reported a lower prevalence of PR changes (2%) than ours and no significant relationship with COVID-19 severity. However, our higher sample population may improve the final result [27]. Similar to recent literature, sinus tachycardia was the most common arrhythmia following viral infections. Additionally, a complete AV block required special cardiac care and a transient pacemaker, changing to 2^nd^ degree wenckebach block and then sinus rhythm [27, 28]. An increase in S and R waves' fragmentation was significantly related to clinical status, with a substantial change in our patients, similar to the literature, who were hospitalized in an ICU [27]. The observed AV blocks—some progressing to complete heart block requiring transient pacing—suggest direct viral injury to conduction pathways or ischemia from systemic inflammation. This finding underscores ECG’s utility in stratifying high-risk pediatric cases, as PR interval abnormalities may precede overt cardiac dysfunction.

The fQRS is either a predictor of various cardiovascular events reflecting non-specific depolarization of the myocardium or an electrocardiographic indicator for myocardial fibrosis. The prognostic importance of fQRS has been studied in patients with myocardial infarction, ischemic and non-ischemic cardiomyopathies, arrhythmogenic right ventricular dysplasia, and hypertrophic cardiomyopathies [28, 29]. In pediatric COVID-19, fQRS may signal subclinical myocardial scar formation due to microangiopathy or cytokine storm-induced damage. Its association with ICU admission reinforces its prognostic value, advocating for fQRS screening to identify children at risk of progressive cardiac involvement, even in the absence of symptoms.

The ST segment is the interval between ventricular depolarization and repolarization. The most significant cause of ST segment abnormalities, whether it is elevation or depression, is myocardial ischemia/infarction. ST depression caused by subendocardial ischemia is most prominent in left precordial leads V4-6, as well as leads I, II, and aVL. According to the Framingham study, the prevalence of ST-T changes was 8.5% in men and 7.7% in women. Additionally, morbidity and mortality of CAD were reported to be two-fold increased in these cases. ST changes indicate heart damage and can be used as a prognostic indicator in these patients. The exact cause of ECG changes in patients with COVID-19 remains unknown. However, several possible mechanisms have been proposed for cardiac involvement associated with COVID-19. Firstly, the angiotensin-converting enzyme (ACE-II), which is detected in high concentrations in the heart and lungs, has been defined as a functional receptor for coronaviruses. Myocardial damage caused by hypoxemia, myocarditis, microangiopathy, MI, and cardiac involvement and damage associated with cytokine storm may have occurred due to a systemic inflammatory response. COVID-19 patients with ischemic parameters (ST-T changes, arrhythmia, and fQRS) should be monitored for the risk of sudden death. In our study, arrhythmia predictors such as fQRS and repolarization changes (*e.g.*, ST-T changes) were independent predictors of cardiac involvement in COVID-19 patients [29, 30]. Sinus tachycardia-the most common arrhythmia-likely reflects systemic inflammation or compensatory mechanisms. These findings suggest that ischemic ECG patterns in pediatric COVID-19 warrant close monitoring for sudden cardiac events, akin to adult populations. ECG changes can be related to multifactorial pathways, including damage from direct viral invasion through ACE2 receptors that disrupt cardiomyocytes and conduction systems, ischemia from hypoxemia from respiratory failure, and microvascular dysfunction from cytokine storm. Early detection of fQRS or ST-T changes could trigger timely interventions (*e.g.*, anti-inflammatory therapies, cardiac monitoring), potentially mitigating long-term sequelae.

## CONCLUSION

The ECG findings, which include ST-T changes, fQRS, and PR interval, are closely linked to the severity of COVID-19. Our study has demonstrated that these ECG findings can be used to predict the presence or absence of myocardial involvement as well as its severity. Furthermore, we have discovered that patients with changes in ST-T and fQRS and PR interval are more likely to experience cardiac involvement, which could result in a poorer prognosis. The study underscores the multifaceted cardiovascular impact of COVID-19 on young patients, emphasizing the need for ongoing cardiovascular evaluations and monitoring. The findings advocate for heightened awareness among healthcare providers regarding the potential for cardiac complications in this demographic, even among asymptomatic individuals. Future research should focus on long-term cardiovascular outcomes in pediatric populations post COVID-19, aiming to establish guidelines for screening and management to mitigate the risk of enduring cardiac issues.

## LIMITATIONS OF THE STUDY

This study was conducted only in hospitals affiliated with Mashhad University of Medical Sciences, which may limit the generalizability of the findings to other populations or geographic regions. Despite examining 764 children, this number may not be sufficient to identify rare patterns or small differences in diverse populations. The study did not explore other causes of heart disease and rhythm disorders, which may affect the interpretation of the results. This is a cross-sectional study, and long-term follow-up of patients to assess long-term cardiac complications was not conducted. Data were extracted from hospital records, which may include recording errors or incomplete information. The study did not investigate the impact of COVID-19 vaccination on ECG findings, which could influence the results. Patients with varying clinical conditions were included in the study, which may affect ECG findings. Patients with varying disease severity were included, which may lead to differences in ECG findings. Although the study found no significant association between age or gender and disease severity, these factors could still act as confounders. Variability in diagnostic methods: COVID-19 diagnosis was based on RT-PCR and imaging, but there may be differences in the accuracy and sensitivity of these methods. ECG interpretation was performed by a trained physician, but subjective differences in interpretation may exist. Patients may have received various medications that could influence ECG findings, but this was not addressed in the study. Differences in nutritional status and general health among patients may affect ECG findings, but these factors were not controlled for in the study. Pediatric patients may have been treated with medications used in COVID-19 treatment, which could potentially affect the QT interval. This is a limitation of the study, and future research should document and account for the use of such medications when analyzing QT intervals.

## RECOMMENDATIONS FOR FUTURE STUDIES

Conducting studies across multiple centers to enhance the generalizability of results. Investigating long-term cardiac complications in children recovering from COVID-19. Examining the impact of other causes of heart disease and rhythm disorders on ECG findings. Assessing the impact of COVID-19 vaccination on ECG findings and cardiac complications. Better control of confounding factors such as nutritional status, general health, and medications used by patients. Future studies should focus on validating fQRS in children, particularly in the context of COVID-19, to confirm its diagnostic and prognostic utility in this age group.

## CLINICAL IMPLICATIONS

The study highlights the utility of ECG as a simple, non-invasive, and cost-effective tool for early detection of cardiac involvement in pediatric COVID-19 patients. This can help clinicians identify myocardial damage or inflammation early, even in asymptomatic cases, allowing for timely intervention and potentially improving clinical outcomes.

ECG findings such as ST-T changes, fragmented QRS (fQRS), and prolonged PR interval can serve as markers for risk stratification. Patients with these abnormalities, especially those requiring ICU admission, may need closer monitoring and more aggressive management to prevent severe cardiac complications.

The presence of ECG abnormalities, particularly fQRS and ST-T changes, may indicate a poorer prognosis in pediatric COVID-19 patients. These findings can help clinicians predict the likelihood of cardiac complications and tailor treatment plans accordingly.

ECG can be used as a screening tool for high-risk pediatric patients, such as those with multisystem inflammatory syndrome in children (MIS-C) or Kawasaki-like disease, to identify hidden cardiac involvement that may not be clinically apparent.

The study suggests that patients admitted to the ICU have a higher prevalence of ECG abnormalities, such as AV node block, arrhythmias, and ST-T changes. This underscores the importance of continuous cardiac monitoring in critically ill pediatric COVID-19 patients to detect and manage life-threatening arrhythmias or conduction disorders promptly.

Given the limited availability and high cost of echocardiography, ECG can serve as a practical alternative for initial cardiac assessment in resource-limited settings. This can reduce the socioeconomic burden on healthcare systems while still providing valuable diagnostic information.

Serial ECG monitoring can help track disease progression and the effectiveness of treatment in pediatric COVID-19 patients with cardiac involvement. Changes in ECG parameters over time can provide insights into the resolution or worsening of myocardial injury.

A significant proportion of pediatric COVID-19 patients in the study were asymptomatic but showed ECG abnormalities. This emphasizes the need for routine ECG screening in children with COVID-19, even in the absence of clinical symptoms, to identify silent myocardial injury.

The findings highlight the importance of a multidisciplinary approach to managing pediatric COVID-19 patients, involving pediatricians, cardiologists, and critical care specialists. Collaboration among these teams can ensure comprehensive care for patients with cardiac complications.

The study provides evidence that can inform the development of clinical guidelines for cardiac monitoring and management in pediatric COVID-19 patients. This is particularly important given the limited data currently available on ECG findings in this population.

The study underscores the need for clinicians to be aware of atypical presentations of cardiac involvement in pediatric COVID-19 patients, such as isolated ECG changes without overt clinical symptoms. This can help prevent missed diagnoses and delayed treatment.

Early identification and management of cardiac involvement in pediatric COVID-19 patients may help prevent long-term complications, such as chronic cardiomyopathy or arrhythmias, improving the overall quality of life for these children.

## Figures and Tables

**Table 1:** Demographic and clinical characteristics.

**Characteristic**	**Value/Percentage**	**Description**
**Total Patients**	**764**	**Pediatric Patients with COVID-19**
**Gender**		
Male	385 (51%)	-
Female	379 (49%)	-
**BMI**		
Median BMI	18.3 ± 18.07	-
**Admission Location**		
General Ward	721 (94.4%)	-
ICU	28 (3.7%)	-
Outpatient	15 (1.9%)	-
**Clinical Classification**		
MISC	198 (25.9%)	-
Kawasaki-like Disease	2 (0.3%)	-
Pulmonary Involvement	97 (12.7%)	-
Gastrointestinal Involvement	15 (2%)	-
**Clinical Status**		
Asymptomatic	451 (59.1%)	Patients with positive PCR but no clinical symptoms

**Table 2:** Laboratory and ECG findings.

**Parameter**	**Value/Percentage**
CKMB > 24 u/liter	10 (1.3%)
TPi Positive	11 (1.5%)
High D-dimer (>500)	63 (78.8%)
ECG Abnormalities	-
Normal Axis (NAX)	755 (98.8%)
Right Axis Deviation (RAD)	5 (0.7%)
Left Axis Deviation (LAD)	1 (0.1%)
Right Conduction Delay	1 (0.1%)
Left Conduction Delay	2 (0.3%)
First-degree AV Block	26 (3.4%)
Second-degree AV Block	1 (0.1%)
Complete Heart Block (CHB)	2 (0.3%)
ST-T Changes	25 (3.3%)
Fragmented S Waves	21 (2.8%)
Fragmented R Waves	13 (1.7%)

**Table 3:** Echocardiography findings.

**Echocardiography Findings**	**Number/Percentage**
**Echocardiographic Changes**	**444 (58%)**
Mitral Valve Insufficiency	
Dilation of Cardiac Chambers	
Pericardial Effusion	

**Table 4:** Association of findings with disease severity.

**Finding**	**Association with Severity**
Prolonged PR Interval	(*p* < 0.05)
Increased QRS Duration	(*p* < 0.05)
AV Node Block and Arrhythmias	(*p* < 0.05)
ST-T Changes	(*p* < 0.05)
Fragmented S and R Waves	(*p* < 0.05)
Echocardiographic Changes	(*p* = 0.02)

**Table 5:** Dysrhythmia distribution on electrocardiogram in studied patients.

**Arrhythymia**	**Frequency**	**Percent**
AVB	29	4.2
Sinus tachycardia	96	12.7
Premature Atrial Contractions (PAC)	3	0.4
Premature ventricular contractions (PVC)	3	0.4
Supraventricular tachycardia (SVT)	1	0.1
Atrial tachycardia (AT)	2	0.3
Sinus bradycardia	10	1.3
Early repolarization	1	0.1
Sinus arrhythmia	1	0.1
Atrial rhythm	2	0.3
Atrial parasystol	1	0.1

**Table 6:** The distribution of fragmentations on cardiac electrocardiogram.

**Fragmentations on ** **Cardiac Electrocardiogram**		**Frequency**	**Percent**
S wave Fragmentation	Normal	724	97.2
	I	2	0.3
	III	1	0.1
	V	5	0.7
	I/II	1	0.1
	II/III	6	0.8
	I/ll/lll	2	0.3
	I/II/avl	1	0.1
	Fragmented S in V2-4	1	0.1
	II,III,AVF,V3-6	1	0.1
	I,II,III,AVF	1	0.1
R wave Fragmentation	Normal	735	97
	II	2	0.3
	III	1	0.1
	V	3	0.4
	III/IVL	2	0.3
	aVL/IVF	1	0.1
	III/LVF	1	0.1
	II/III/I/AVF	3	0.4

## Data Availability

The data that support the findings of this study are available from the corresponding author upon reasonable request.

## LIST OF ABBREVIATIONS

ACE-II: Angiotensin-Converting Enzyme 2
AVB: Atrioventricular Block
BMI: Body Mass Index
CAD: Coronary Artery Disease
CHB: Complete Heart Block
CKMB: Creatine Kinase-MB
ECG: Electrocardiogram
fQRS: Fragmented QRS
ICU: Intensive Care Unit
LAD: Left Axis Deviation
MISC: Multisystem Inflammatory Syndrome in Children
NAX: Normal Axis
PAC: Premature Atrial Contraction
PVC: Premature Ventricular Contraction
RAD: Right Axis Deviation
RT-PCR: Reverse Transcription Polymerase Chain Reaction
SARS-CoV-2: Severe Acute Respiratory Syndrome Coronavirus 2
SPECT: Single-Photon Emission Computed Tomography
SVT: Supraventricular Tachycardia
TPi: Troponin I
WHO: World Health Organization

## STANDARDS OF REPORTING

STROBE guidelines were followed.
